# Primary pancreatic plasmacytoma: a rare case report

**DOI:** 10.1186/s12876-017-0729-z

**Published:** 2017-12-20

**Authors:** Tao Lu, Hong Pu, Gaoping Zhao

**Affiliations:** 1 Department of Radiology, Sichuan Academy of Medical Science & Sichuan Provincial People’s Hospital, 32 West Second Section, First Ring Road, Chengdu, Sichuan 610072 China; 2 Department of Gastrointestinal Surgery, Sichuan Academy of Medical Science & Sichuan Provincial People’s Hospital, 32 West Second Section, First Ring Road, Chengdu, Sichuan 610072 China

**Keywords:** Pancreas, Plasmacytoma

## Abstract

**Background:**

Extramedullary plasmacytoma is a very rare tumor derived from plasma cells and found outside the bone marrow. Most have been identified in patients with the more aggressive anaplastic form of the disease. Only a few cases of primary pancreatic plasmacytoma have been reported.

**Case presentation:**

We present a case of a 56-year-old man in whom a pancreatic mass was found incidentally. The lesion was determined to be a pancreatic plasmacytoma after distal pancreatectomy. There are no indications of clinical, laboratory or imaging findings of multiple myeloma nor any association with plasmacytoma in any other places, so the diagnosis of primary pancreatic plasmacytoma was made.

**Conclusion:**

Primary pancreatic plasmacytoma is rare and the diagnosis is difficult before surgery.

## Background

Extramedullary plasamacytomas are plasma cell tumours that present outside the bone marrow. Most commonly, they have been found in the upper respiratory tract but the primary pancreatic plasmacytoma is rare. Here we report a case of primary pancreatic plasmacytoma that was found incidentally.

## Case presentation

**Fig. 1:**
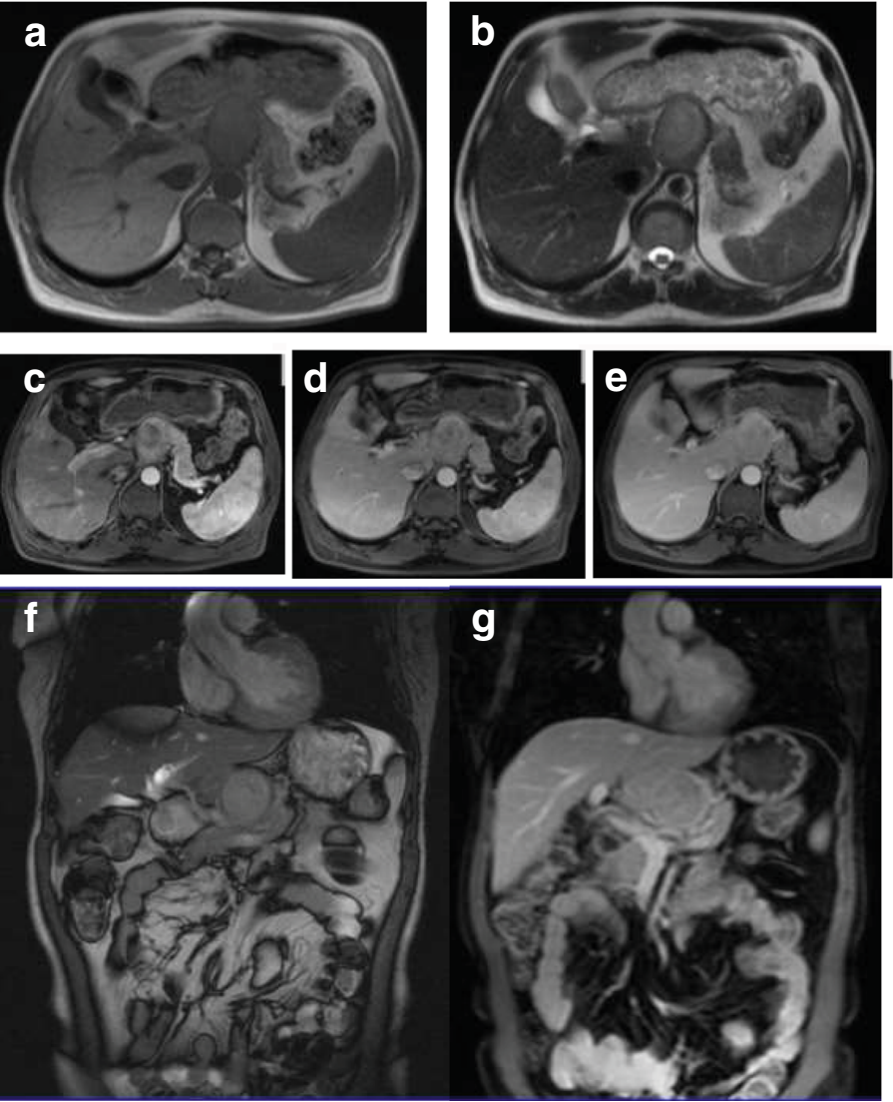
**a**-**g** MRI images showed a mass that was hypointense on T1WI and mildly hyperintense on T2WI in the pancreas with gradual, moderate, heterogeneous enhancement. The caudate lobe of the liver was compressed, and the mass had a poor margin with respect to the celiac trunk, abdominal aorta and the right crus of diaphragm

**Fig. 2:**
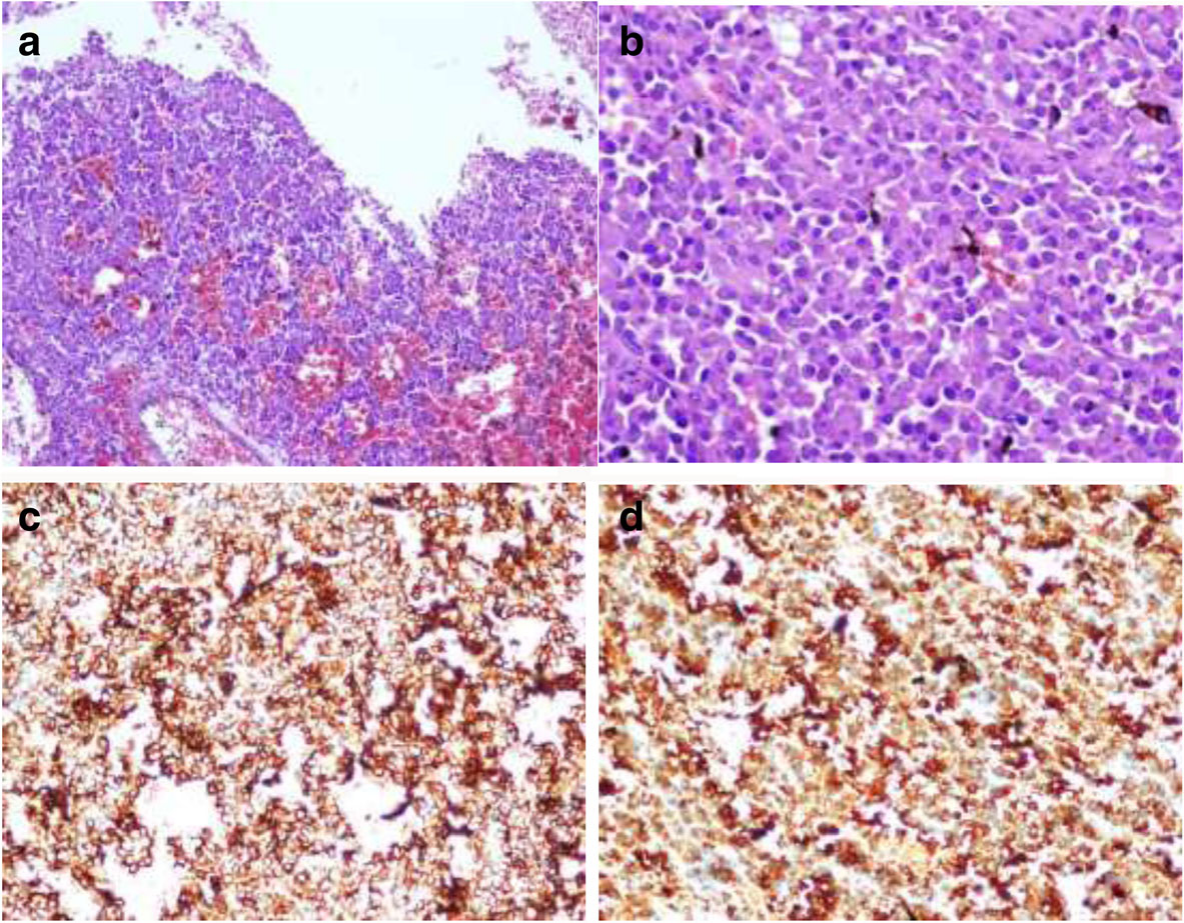
**a** and **b** Hematoxylin and eosin stain. Original and magnification ×100 and ×400. The plasma cell size and nuleus are polymprphic. **c** and **d** The neoplastic cells are positive for CD138 and kappa light chain

A 56-year-old man complained of chest and back pain and discomfort after activity for over 20 days with no other symptoms or observed signs. Routine blood test suggested infection (WBC 10.12 × 10^9^/L, NEU 9.41 × 10^9^/L, NEU ratio93%) and identified anemia (RBC 3.26 × 10^12^/L, haemoglobin 98 g/L). Serum bilirubin level and lactate dehydrogenase were elevated. Electrocardiogram revealed myocardial ischemia. After admission, a routine abdominal utrasonogram was obtained and it detected a hypoechoic mass in the pancreas near the celiac trunk. MRI (Fig. 1a-g) confirmed a 5.1 × 3.8 cm circumscribed mass that was hypointense on T1WI and mildly hyperintense on T2WI in the body of the pancreas. The mass protruded outside the profile of the pancreas and compressed the caudate lobe of the liver. The peripancreatic fat was intact with absence of bile duct dilation, but the mass had a poor margin with respect to the celiac trunk, abdominal aorta and the right crus of diaphragm. The mass showed moderate, heterogeneous enhancement on dynamic contrast enhancement images. However, tumour markers including CA125, CA199, CEA, and AFP were all within the normal limits. Resection of the body and tail of the pancreas was performed. A hard mass measuring 10 cm in diameter was palpated in the body of the pancreas. The mass was tightly adhered to the celiac trunk, abdominal aorta, inferior vena cava and splenic vein and was relatively immobile. Pathological and immnunohistological staining (Fig. 2a-d) was positive for kappa light chain, CD138 and vimentin, and negative for lambda light chain and CD38. We confirmed that there were no osseous or other identifiable lesions by CT, radiography and FDG-PET examinations, and bone marrow puncture showed normocellularity. Results from serum protein electrophoresis and urine Bence-Jones protein electrophoresis were all normal. Therefore, this case was diagnosed as primary pancreatic plasmacytoma. Discharge from the hospital occurred 1 month after surgery. There was no evidence of bone marrow or involvement of extramedullary sites during 2 years of follow-up. During follow-up, the patient underwent coronary artery bypass graft because of severe coronary atherosclerotic heart disease and unstable angina. The patient did well after this surgery.

## Discussion and conclusions

Extramedullary plasmacytoma is a kind of rare neoplasm, occurring in less than 5% of plasma cell tumors, and is usually diagnosed after multiple myeloma of the bone marrow [1]. Although extramedullary lesions can involve any tissue or organ, the most common site is the submucosal lymphoid tissue of the upper respiratory tract. Only 10% of extramedullay plasmacytomas occur in the gastrointestinal tract, where they are detected most often in the liver, spleen, or stomach [2, 3]. Pancreatic involvement is rare, being found in approximately 2.3% of autopsies [4]. After conducting a systematic Pubmed search, we found about 64 cases reports of pancreatic plasmacytoma; most of these cases were associated with myelomatosis with spread from other extra-osseous or bony sites. Only a few cases of primary pancreatic plasmacytoma have been previously reported in the English language literature [2, 5–8]. In our case, the pancreatic mass was found incidentally; no indications of clinical, laboratory or imaging findings of multiple myeloma nor any association with plasmacytoma in any other places, which confirmed it was a case of primary pancreatic plasmacytoma.

Although plasmacytoma can develop in any part of the pancreas; the most frequent site of the tumor is the pancreatic head from the reported cases. Patients are always demonstrated with abdominal pain and obstructive jaundice due to the compression of the common bile duct. Diffuse enlargement or a mass in the tail of the pancreas have also been reported [2, 3, 9]. Only 3 cases involving the body of the pancreas have been reported previously [1, 10, 11]. Masses are more likely to be detected incidentally when located in the body because masses located in the head of the pancreas are more likely to compress the biliary tree, leading to observable signs and symptoms related to obstruction. In our case, the patient was admitted initially for suspected myocardial ischemia. The mass in the pancreatic body was detected incidentally by ultrasonography and confirmed by MRI. In the absence of any symptoms related to multiple myeloma or any manifestations of mass effect, it may be very difficult to detect the tumour at an early stage. Whether the patient would have eventually developed such manifestations or still had such a favourable post-surgical outcome is uncertain but it is likely the patient benefitted greatly from early detection.

Imaging modalities for detecting the disease usually include ultrasonography, CT and MRI, endoscopic ultrasonography (EUS) and EUS guided fine-needle aspiration (FNA). Ultrasonography typically demonstrates a heterogeneous focal mass that is hypoechoic relative to the normal parenchyma [12]. CT is a widely used method for assessing pancreatic masses. The CT appearance of pancreatic plasmacytoma is well established and is typically described as a multilobular homogeneous solid tumour that is hypodense as compared to the pancreatic parenchyma and with homogeneous intravenous contrast enhancement [13]. MRI is often better in demonstrating pancreatic masses than CT. MRI features include pancreatic enlargement with a lobulated contour, lower signal intensity than that of the liver on T1WI, and diffusely increased signal intensity on T2WI with heterogeneous enhancement [3, 14]. In our case, MRI findings were comparable with those of previous reports. The tumour was hypointense on T1WI and mildly hyperintense on T2WI. Gradual, moderate, heterogeneous enhancement of the tumour was observed on dynamic contrast enhancement images. MRI is also better at revealing adjacent structure infiltration, destruction of the pancreatic or common bile duct or involvement of the peripancreatic vasculature. As in our case, MRI showed the tumour had a poor margin with respect to the celiac trunk, abdominal aorta and the right crus of diaphragm, but without the involvement of the pancreatic or bile duct. The size of the tumour was much bigger when palpated during surgery than measured on MRI images, this discrepancy may be because the surgeons palpated not only the tumour itself, but also part of the pancreatic body and the adjacent structure. EUS and EUS-FNA are being increasingly used in the diagnosis of pancreatic tumours. They are able to provide an accurate visualization and histopathologic diagnosis. Studies have shown that the overall accuracy for EUS-FNA ranges between 71% and 90% in cases of pancreatic tumours [15]. However, EUS is very operator-dependent and its value varies widely with locally available expertise [16, 17]. EUS-FNA also has risks of pancreatitis, bleeding, and perforation.

The final diagnosis of extramedullary plasacytoma relies on the demonstration of a monoclonal plasma cell infiltration without the present of myeloma [18]. In order to confirm the monoclonality, it is necessary to demonstrate monoclonal kappa or lambda light chains, heavy chains, or plasma cell markers, such as CD38 from immunohistological examination [19]. In our case, the tumour cells demonstrated strong positivity for plasma cell marker CD138 and kappa light chain in the absence of lambda light chain staining in keeping with the monoclonal nature of myeloma.

The radiologic features of extramedullary plasmacytoma are non-specific and may resemble typical findings in other pancreatic neoplasms including adenocaricinoma, neuroendocrine tumors, lymphoma and metastases. Pancreatic adenocarcinoma is originated from the ductal epithelium and is hypovascular. The tumor often (66%) occurs in the pancreas head and represents approximately 85% of pancreatic masses. It is usually iso- or hypodense on CT images; and is genearlly hypointense on T1WI and slightly hyperintense on T2WI on MRI images. Due to the hypovascularity, the tumor demonstrates only minimal enhancement compared with obvious enhancement of the normal pancreatic parenchyma. Atrophy of the distal pancreas, dilation of the pancreatic duct and bile duct, invasion of the adjacent vasculature and lymph nodes, and liver metastases could also be detected in pancreatic adenocarcinoma. Pancreatic neuroendocrine tumours are rare pancreatic tumours that originate from pluripotent stem cells in the ductal epithelium of the pancreas [20]. The tumour is iso- to hypo- dense on CT scan, relatively hypointense on T1WI and generally hyperintense on T2WI. Functional NETs are generally small (1-2 cm) and manifest as well-defined, hypervascular lesions owing to their rich capillary network. Non-functioning tumours, on the other hand, are relatively larger in size (mean 4 cm), are often well-defined and encapsulated, and show heterogeneous enhancement. Primary pancreatic lymphoma is also rare and usually an extranodal manifestation of B cell non-Hodgkin’s lymphoma. It can either be localized or appeared as a well-defined focal mass with minimal but homogeneous enhancement from contrast-enhancement CT, or an infiltrative manifestation replacing the whole pancreas and simulating acute pancreatitis [21, 22]. Significant peripancreatic lymph node enlargement with disseminated lymphadenopathy, usually can be found. The pancreas is an unusual site for metastases. They are typically seen with advanced disease in such condition. They can be localized, multifocal, or show diffuse enlargement on CT [23]. The enhancement pattern of the metastatic lesion are always similar to that of the primary tumour [24]. On MRI, they are typically hypointense on T1WI and enhance avidly.

There appears to be no standardized treatment for extramedullary plasmacytoma of the pancreas. Surgery, radiotherapy and chemotherapy with haematopoietic stem cell transplantation have all been employed. For solitary plasmacytoma, distal pancreatectomy of the body or tail of the pancreas can be performed in good surgical candidates. Incidental cases may undergo pancreatic resection typical for pancreatic lesions with subsequent identification of plasma cells [2]. However, owing to the often systemic nature of the disease and the radical nature of these surgical procedures, surgeries are not commonly performed. On the other hand, since the plasma cell tumours are highly radiosensitive, radiation therapy has also been suggested to be the treatment of choice [1]. When plasmacytomas are secondary, chemotherapeutic agents are also commonly used, combined or not with radiotherapy. In eligible patients, the standard of care of plasm cell myeloma is steroid-chemotherapy combinations followed by autologous haematoopoietic stem cell transplantation (auto- HSCT) [10]. Consequently, the course for extramedullary plasmacytoma not related to multiple myeloma is more favourable than that of multiple myeloma or solitary plasmacytoma of the bone. In our patient with a solitary plasmacytoma in the body of the pancreas, resection of the pancreatic body and tail was performed without radiotherapy of chemotherapy. The patient’s overall prognosis was good during the follow-up.

In conclusion, this report represents a rare case of primary pancreatic plasmacytoma located in the body of the pancreas. The patient demonstrated no relevant symptoms or evidence of multiple myeloma, so it was difficult to diagnose before surgery. Pancreatic resection was highly successful for this patient with a solitary pancreatic tumour.

## Abbreviations

AFP: α-fetoprotein
CA125: Carbohydrate antigen 125
CA199: Carbohydrate antigen 199
CD138: Cluster of Differentiation 138
CD38: Cluster of Differentiation 38
CEA: Carcinoembryonic Antigen
CT: Computed tomography
EUS: Endoscopic ultrasonography
FDG-PET: ᅟ
FNA: Fine-needle aspiration
MRI: Magnetic resonance imaging
NET: Neuroendocrine tumors
NEU: Neutrophils
RBC: Red blood cell
T1WI: T1 weighted images
T2WI: T2weighted images
WBC: White blood cell
